# Pomegranate juice and punicalagin-mediated chemoprevention of hepatocellular carcinogenesis via regulating miR-21 and NF-κB-p65 in a rat model

**DOI:** 10.1186/s12935-022-02759-9

**Published:** 2022-11-02

**Authors:** Aya M. Hussein, Nadia M. El-Beih, Menha Swellam, Enas A. El-Hussieny

**Affiliations:** 1 Zoology Department, Faculty of Science, Ain Shams University, Khalifa El‑Maamon St, Abbasiya Sq, Cairo, 11566 Egypt; 2 Biochemistry Department, National Research Centre, Dokki, Egypt

**Keywords:** Apoptosis, HCC, Hepatic antioxidants, miR-21, NF-κB-p65, TNF-α, Pomegranate juice, Punicalagin

## Abstract

**Background:**

Hepatocellular carcinoma (HCC) is the most common neoplasm among primary liver malignancies, accounting for 70%–85% of total liver cancer cases worldwide. It is also the second-leading cause of cancer-related death worldwide. Recent research has investigated naturally occurring products high in polyphenolic compounds in the regression and prevention of HCC. This study investigated the chemoprevention effects of pomegranate juice (PJ) and punicalagin (PCG) against diethylnitrosamine (DENA)-induced hepatocarcinogenesis in male albino rats.

**Methods:**

Animals were randomized into six groups and treated for 11 weeks as follows: group 1 was a negative control group, group 2 was treated orally with 10 mL PJ per kilogram body weight (kg bw), group 3 was treated orally with 18.5 mg PCG/kg bw, and groups 4–6 were injected with an intraperitoneal dose of DENA (50 mg/kg bw) weekly beginning in the third week. Group 4 was a HCC control (DENA-treated group), group 5 was HCC + PJ, and group 6 was HCC + PCG.

**Results:**

PJ antagonized DENA-induced elevations of ALAT, TNF-α, NF-κB-p65, GST, MDA, and NO and restored total protein, IL-10, SOD, and CAT levels. Moreover, PJ resulted in downregulation of miR-21, Bcl-2, and Bcl-XL and an upregulation of caspase-3 and Bax mRNA expressions. These chemoprevention effects of PJ also alleviated the hepatic preneoplastic lesions induced by DENA. Although PCG treatment induced some modulation in DENA-treated rats, it did not show potent chemoprevention activity and induced some side effects.

**Conclusion:**

Both of PJ and PCG downregulated miR-21 expression and triggered apoptosis. However, PJ was more effective than pure PCG in alleviating the hepatic antioxidant defense state and the inflammatory status. So, PJ was superior in prevention of DENA-induced hepatocellular carcinogenesis in rats than pure PCG.

**Graphical Abstract:**

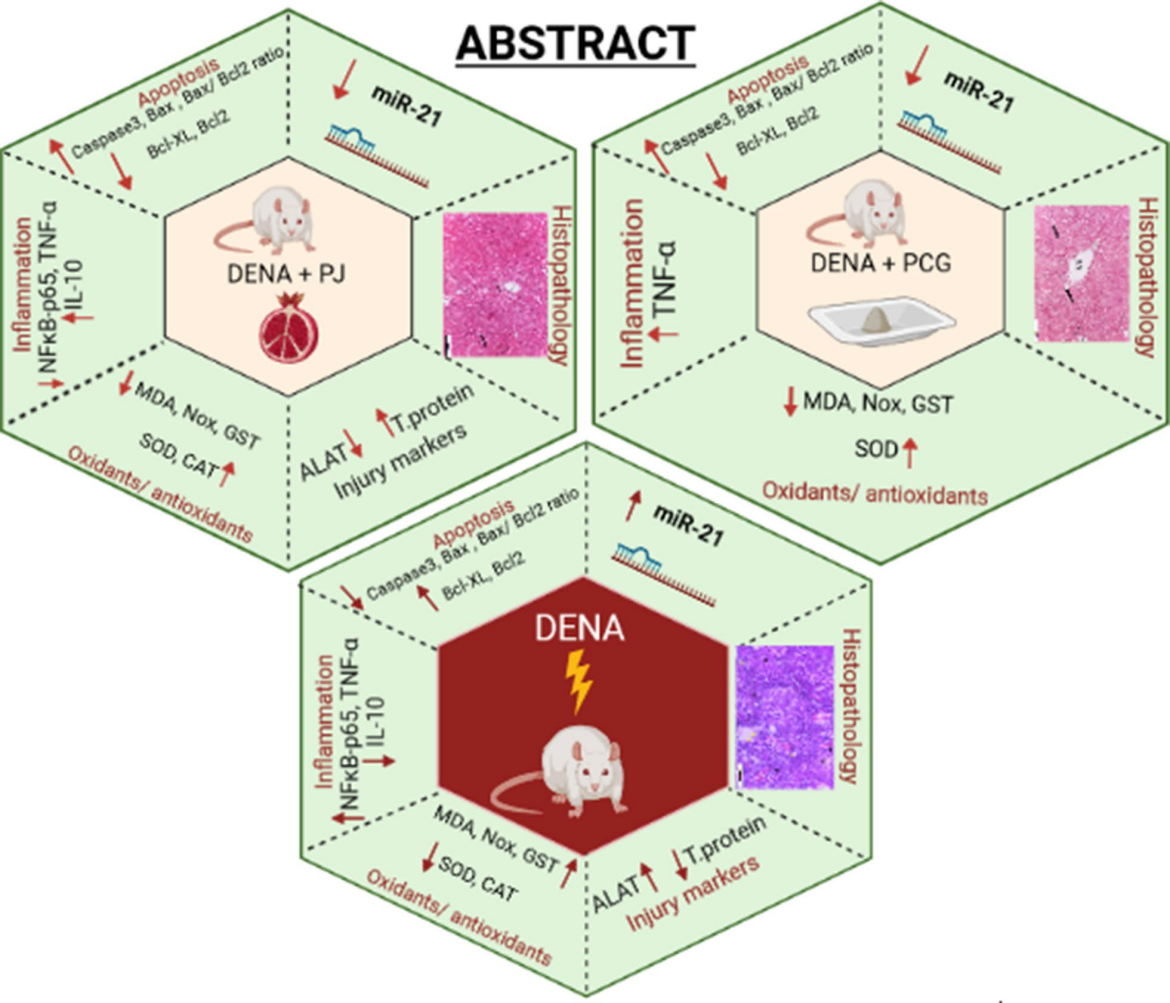

## Background

Hepatocellular carcinoma (HCC) is the most common neoplasm among primary liver malignancies. It is the sixth most prevalent malignancy and the second-leading cause of cancer-related death worldwide, accounting for 70–85% of total liver cancer cases [1]. It usually occurs as a result of underlying liver disease and is frequently associated with liver fibrosis or cirrhosis arising from persistent liver injuries [2]. The lack of early diagnosis and high recurrence rates for HCC stages have drawn research attention toward studying the molecular mechanisms of this disease [3]. Disturbances in the hepatic antioxidant and oxidative stress and inflammatory systems have been implicated in the development and progression of cirrhosis to induce liver cancer [4, 5]. Additionally, overexpression of inflammatory cytokines linked to overproduction of reactive oxygen species (ROS) could inhibit apoptosis, presumably by activating the nuclear factor (NF)-κB-dependent pathway [6].

MicroRNAs (miRNAs), small noncoding single-strand RNA molecules (18–24 nucleotides), are involved in the regulation of gene expression at the post-transcriptional and translational levels [7]. The stability of miRNAs in the circulation from both healthy and diseased tissues makes them ideal biomarkers for different diseases. Successful miRNA-based therapy would certainly lead to improved efficacy [8]. MiR-21, one of the first identified mammalian miRNAs, has been identified as an oncogenic miRNA that is overexpressed in various human malignancies, including liver cancer [9]. MiR-21 upregulation has been reported in cirrhosis—the end-stage fibrosis—which is a prevalent preneoplastic condition linked to hepatocarcinogenesis [10].

Target-based therapy is widely thought to represent the future of cancer treatment, and so the development of inhibitors of HCC-signaling pathways and their upstream activators has generated considerable interest [11]. Thus, scientific interest in cancer management has been directed toward the utilization of natural products high in polyphenolic compounds due to their reported chemopreventive potential, and in an effort to prevent toxicities induced by chemicals, drugs, and carcinogenic xenobiotics. Furthermore, nearly 70% of all cancer medications used currently is derived from natural products [12].

Pomegranate (*Punica granatum* L.) fruit has become increasingly popular as a functional food of potential health benefits due to its ability to counter oxidative stress and reduce inflammatory mediators [13]. For cancer prevention and therapy, it is critical to maintain healthy physiological condition by balancing free radicals and antioxidants [14]. Among the bioactive ingredients in pomegranates is a group of polyphenols called punicalagins (PCGs), which are unique to the pomegranate. Pomegranate juice (PJ) has antioxidative, anti-inflammatory, anti-proliferative, and pro-apoptotic actions that exceed those observed with their isolated active components, suggesting that therapeutic strategies that do not rely on pure single agents could be developed [15, 16]. In this regard, this study evaluated and compared the chemoprevention effects of PJ and PCG in a hepatocellular carcinogenesis rat model. Moreover, this study tested a potential signaling pathway by surveying oxidative stress, inflammation, and apoptosis with particular focus on miR-21 expression.

Diethylnitrosamine (DENA), an environmental and dietary hepatocarcinogen, is a DNA-alkylating agent widely used to induce liver cancer in animal models with a high success rate and with similarities to human HCC. Recently, DENA-induced rodent HCC models have been established to elucidate the pathogenesis, prevention, and treatment of liver cancer, including miRNA functions, antitumor effects of drugs and identification of biomarkers, and therapeutic targets [17].

## Materials and methods

### Chemicals and natural products

Diethylnitrosamine (DENA) was purchased from Sigma-Aldrich (St. Louis, MO, USA). Fresh pomegranate fruits, free of obvious defects, were purchased from Dina farms (Cairo-Alexandria Desert Road, Egypt) and kindly were authenticated by our colleagues in the Botany Department.; fruits were manually washed and peeled, and separated arils were processed using a commercial blender to obtain PJ, and the juice was filtered and was provided orally to the animals within 1 week after squeezing. PCG (purity ≥ 80% HPLC) was purchased from Xi’an Rongsheng Biotechnology Co., Ltd. (82 Keji Road, Xi’an Hi-tech Zone, China).

### Experimental animals

Adult male Wistar albino rats (*Rattus norvegicus*; 200 ± 10 g) were purchased from the Nile Company for pharmaceuticals and chemical industries (Cairo, Egypt). The animals were housed in suitable cages and acclimatized to laboratory conditions for 1 week before the experiments began. Rats were provided with fresh tap water and standard rodent food pellets (Agriculture-Industrial Integration Company, Giza, Egypt). The animals were humanely treated in accordance with WHO guidelines for animal care, and the study design was approved by the Ain Shams University Research Ethics Committee (4/ 2018).

### Induction of hepatocellular carcinogenesis

A hepatocellular carcinogenesis model was induced according to Cheng et al*.* [18] with some modifications, starting at the beginning of 3rd week; small doses of DENA (50 mg per kilogram body weight, once weekly) were injected intraperitoneally for 9 consecutive weeks. At the end, liver histopathology examination revealed early-stage HCC as demonstrated by dysplastic lesions, which have been described as possible progenitor lesions to HCC.

### Experimental design and groups of animals

After 1 week of acclimatization, animals were randomly divided into six groups and treated for 11 consecutive weeks (77 days) as follows:

1. Group I (control group): the animals received a basal diet and fresh water and housed in the same conditions as the treated groups.
2. Group II (PJ group): 10 mL of PJ/kg bw, administered orally daily according to the previous studies of Ahmed et al. [19, 20].
3. Group III (PCG group): 18.5 mg of PCG/kg bw, administered orally daily according to our previous study El-Beih et al. [21].
4. Group IV (HCC control group): after induction of HCC, the animals served as a reference group for the DENA-treated groups.
5. Group V (HCC + PJ group): the animals were injected with an intraperitoneal (i.p.) dose of DENA (50 mg/kg bw, weekly) beginning in the 3^rd^ week until the end of the experiment and received 10 mL PJ/kg bw, administered orally daily, from the first day of the experiment until the end of the 11th week.
6. Group VI (HCC + PCG group): the animals were injected with an i.p. dose of DENA (50 mg/kg bw, weekly) beginning in the 3^rd^ week until the end of experiment and received 18.5 mg PCG/kg bw, administered orally daily, from the first day of the experiment until the end of the 11th week.

### Blood and tissue sampling

At the end of the experiment (day 78), the animals were subjected to light diethyl ether anesthesia before sacrificing. Part of the blood was collected into serum sep vacuette tubes and was then centrifuged in a cooling centrifuge (IEC centra-4R, International Equipment Co., Needham Heights, MA, USA) for 15 min at 4000 rpm and 4 °C to obtain serum. The serum samples were preserved at − 80 °C until they were used for biochemical analysis. Another part of the blood was collected into serum sep clot activator, left at room temperature (RT) for 30 min, and was then centrifuged at 10,000 rpm at 4 °C for 15 min. This part was used for detection and assessment of targeted miRNA.

Immediately after sacrificing the animals, the liver was separated from the body, rinsed in 0.9% saline, weighted, and divided into three parts: (**a)** the first part was depressed in a 1-mL Qiazol solution to be used for the molecular assessments of different genes; (**b)** another part was stored at − 80 °C until it was used for biochemical assessment of oxidant/antioxidant markers; and (**c)** the last part was depressed in an appropriate fixative (10% formalin) for histopathological examination.

### Serum ALAT and total protein

The activity of serum alanine aminotransferase (ALAT) and the level of serum total protein were estimated using commercial kits (Giza, Egypt).

### Inflammatory markers

Sandwich ELISA kits were used for quantitative estimation of serum interleukin-10 (IL-10) and TNF-α (Koma Biotech Inc., Seoul, Korea), as well as hepatic NF-κB-p65 (Elabscience, Biotech Inc., USA) concentrations, according to the manufacturers’ instructions.

### Hepatic antioxidants and oxidative stress markers

Liver tissue homogenate was used for analysis of malondialdehyde [MDA; 22], nitric oxide [NO; 23], catalase [CAT; 24], superoxide dismutase [SOD; 25], and glutathione S-transferase [GST; 26].

### Measurement of apoptotic gene expression in liver cells

Total ribonucleic acid (RNA) was extracted from liver tissues using Triazol extraction reagent (Bioflux Technology Co., China) following the manufacturer’s instructions. Reverse transcription of total RNA to first-strand complementary deoxyribonucleic acid (cDNA) was then carried out using Agilent Sure-Cycler 8800 (Agilent Technologies, Santa Clara, California) at 37 °C for 10 min. Gene expressions of Caspase-3, Bax, Bcl-2, and Bcl-XL were quantified by real-time polymerase chain reaction (qPCR), and the expression of hepatic glyceraldehyde-3-phosphate dehydrogenase (GAPDH, a house-keeping gene) was employed for normalizing the expressions of the tested genes.

The qPCR was performed with specific primers obtained from Sigma-Aldrich for caspase-3 (sense, 5-CTGGACTGCGGTATTGAGAC-3; antisense, 5-CCGGGTGCGGTAGAGTAAGC-3), Bax (sense, 5-GACACCTGAGCTGACCTTGG-3; antisense, 5-GAGGAAGTCCAGTGTCCAGC-3), Bcl-2 (sense, 5-CAAGCCCGGGAGAACAGGGTA-3; antisense, 5-CCCACCGAACTCAAAGAAGGC-3) and Bcl-XL (sense, 5-TCAATGGCAACCCTTCCTGG-3; antisense, 5-ATCCGACTCACCAATACCTG-3), and GAPDH (sense, 5-ACCACAGTCCATGCCATCAC-3; antisense, 5-TCCACCACCCTGTTGCTGTA-3). All PCR reactions were performed using Maxima SYBR Green qPCR Master Mix (GenedireX, USA) according to the manufacturer’s instructions and were carried out using the Agilent Mx3005Pro qPCR system (Agilent Technologies Company, Santa Clara, CA, USA). The amplification conditions were one cycle for initial denaturation at 95 °C for 10 min, followed by 40 cycles of denaturation (15 s at 95 °C), annealing (60 s at 60 °C), and a final extension (20 s at 72 °C). Differences in gene expression between groups were calculated using the △△cycle threshold (Ct) method [27].

### Measurement of serum circulating miR-21 expression

Total microRNA from the serum samples was isolated using the miRNeasy Mini Kit (Cat number #217,004; Qiagen, Hilden, Germany) in line with the manufacturer’s instructions. Reverse transcription of miRNA was carried out using Agilent SureCycler 8800 (Agilent Technologies, Santa Clara, California) with the MiScript II reverse transcription kit (Cat number # 218,160, Qiagen, USA). Finally, qPCR was performed using the MiScript primer assay (Cat number #218,300, Qiagen, USA), miRNA-21 (Hs_miR-21_2, MS00009079), and the reaction was tested using MiScript SYBR Green PCR kit (Cat number # 218,073; Qiagen, USA). Further, RNU-16 (Hs_RNU-2_11, MS00033740) was used as an endogenous control to normalize the expression level of the investigated miRNA. The relative expressions of miRNA were calculated according to Livak and Schmittgen [27].

### Histopathological examination

Liver specimens previously fixed in 10% formalin were embedded in paraffin, sectioned at 5 µm, and stained with hematoxylin and eosin.

### Statistical analysis

Statistical analysis was performed with one-way analysis of variance (ANOVA), followed by Tukey’s multiple comparison test, using GraphPad Prism version 4.03 for Windows (GraphPad Software, Inc., San Diego, CA, USA) for determining statistical significance. *P* values of < 0.05, < 0.01, and < 0.001 were considered statistically significant, highly significant, and very highly significant, respectively. Data are expressed as mean ± standard error of the mean (SEM).

## Results

### Chemoprevention effects of PJ versus PCG on body weight, liver weight, and biochemical indices in control and DENA-treated rats

The HCC control group showed a significant decrease (*P* < 0.05–0.001) in body weight, liver weight, and serum total protein levels and a significant increase (*P* < 0.05–0.001) in liver relative weight and the ALAT activity compared to the control group (Table 1). Although oral treatment of the DENA-treated groups with either PJ or PCG significantly increased (*P* < 0.001) body weight, only PJ treatment of the DENA-treated group led to a significant decrease in relative liver weight (*P* < 0.01). Treatment of the DENA-treated group with PCG non-significantly decreased (*P* > 0.05) relative liver weight as compared with the HCC control group. Despite the non-significant effect of PCG oral treatment on serum total protein levels in DENA-treated rats (*P* > 0.05), a significant increase was reported in total protein levels in the DENA-treated group that received PJ (*P* < 0.001) as compared to the HCC control group. While treatment of DENA-treated rats with PJ led to a significant decrease in serum ALAT (*P* < 0.001), treatment of DENA-treated rats with PCG did not significantly affect serum ALAT activity (*P* > 0.05) as compared with the HCC control group.

**Table 1:** Body-weight, Liver weight, Liver relative weight and biochemical indices of control and carcinogenic male albino rats treated with either pomegranate juice (PJ) or punicalagin (PCG)

Group	Non-carcinogenic groups			Carcinogenic groups		
	Control	PJ (10 mL/ kg b.w)	PCG (18.5 mg/ kg b.w)	HCC (50 mg DENA/ kg b.w)	HCC (50 mg DENA/kg b.w) + PJ(10 mL/ kg b.w)	HCC (50 mg DENA/ kg b.w) + PCG (18.5 mg/ kg b.w)
Body weight (g)	77.74 ± 2.72	65.62 ± 2.22^*^	63.10 ± 2.45^**^	21.40 ± 1.91^***^	47.80 ± 0.58^***###^	41.60 ± 3.37^***###^
Liver weight (g)	7.07 ± 0.10	7.30 ± 0.14	6.54 ± 0.24	6.06 ± 0.20^*^	6.68 ± 0.24	6.13 ± 0.21^*^
Liver relative weight (g/ 100 g bw)	2.63 ± 0.25	2.89 ± 0.06	2.64 ± 0.07	3.25 ± 0.08 ^*^	2.32 ± 0.03^##^	2.68 ± 0.19
ALAT (U/ mL)	6.83 ± 0.76	5.00 ± 0.48	11.45 ± 1.05^*^	19.21 ± 1.38^***^	8.23 ± 0.67^###^	16.44 ± 0.64^***^
Total protein (g/ dL)	8.58 ± 0.34	7.44 ± 0.28	7.08 ± 0.30^*^	6.17 ± 0.17^***^	8.37 ± 0.30^###^	6.77 ± 0.38^**^

Data are presented as means and their standard error of means (n = 5 in each group). ALAT alanine aminotransferase; HCC hepatocellular carcinogenesis control group.

*, ** and ***: *P* < 0.05, *P* < 0.01 and *P* < 0.001 (compared to control group)

^**##**^and ^**###**^: *P* < 0.01 and *P* < 0.001 (compared to HCC control group)

### Chemoprevention effects of PJ versus PCG on hepatic oxidative stress and antioxidant status in control and DENA-treated rats

HCC control rats showed a significant increase (*P* < 0.05) in the activity of liver GST as compared with the control group. However, oral treatment of the DENA-treated rats with either PJ or PCG led to a significant decrease in the hepatic activity of GST (*P* < 0.05) as compared with the HCC control group. Liver CAT and SOD activities significantly decreased (*P* < 0.05–0.001) in the HCC control group as compared with the control group. However, oral treatment of the DENA-treated rats with PJ led to significant increases in the activity of liver CAT and SOD (*P* < 0.01–0.001), while DENA-treated rats that received PCG orally showed a significant increase in liver SOD activity (*P* < 0.001) accompanied with a non-significant increase (*P* > 0.05) in liver CAT activity as compared with the HCC control group. On the other hand, the HCC control rats showed a significant increase (*P* < 0.01) in liver MDA and NO levels as compared with the control group. However, these elevations showed significant decreases in the DENA-treated groups treated with either PJ (*P* < 0.01) or PCG (*P* < 0.001; Table 2).

**Table 2:** Antioxidant defence system and oxidative stress markers in liver tissues of control and carcinogenic male albino rats treated with either pomegranate juice (PJ) or punicalagin (PCG)

Group	Non-carcinogenic groups			Carcinogenic groups		
	Control	PJ (10 mL/ kg b.w)	PCG (18.5 mg/ kg b.w)	HCC (50 mg DENA/ kg b.w)	HCC (50 mg DENA/kg b.w) + PJ(10 mL/ kg b.w)	**HCC** **(50 mg DENA/ kg b.w)** ** + ** **PCG (18.5 mg/ kg b.w)**
MDA level (μM/mg protein)	80.44 ± 3.82	74.54 ± 3.36	92.19 ± 4.06	117.76 ± 0.06^**^	82.25 ± 5.80^##^	74.65 ± 5.68^**###**^
NO level (μmol/ L)	18.74 ± 1.14	19.34 ± 0.81	19.86 ± 0.39	23.65 ± 0.89^***^	19.19 ± 0.31^##^	17.34 ± 0.30^**###**^
GST activity (U/ mg protein)	8.55 ± 0.51	10.83 ± 0.57	10.05 ± 1.08	12.39 ± 0.92^*^	8.25 ± 0.52^#^	8.64 ± 1.15^**#**^
SOD activity (U/ mg protein)	79.79 ± 2.78	87.16 ± 4.62	75.23 ± 4.59	39.95 ± 2.01^***^	68.26 ± 2.77^###^	65.21 ± 1.88^*###^
CAT activity (U/ mg protein)	91.04 ± 3.58	79.39 ± 4.93	75.64 ± 1.52	69.19 ± 2.98^*^	94.32 ± 4.61^##^	75.24 ± 5.94

Data are presented as means and their standard error of means (n = 5 in each group). CAT catalase, GST glutathione S-transferase, HCC hepatocellular carcinogenesis control group, MDA malondialdehyde, NO nitric oxide, SOD superoxide dismutase

*, ** and ***: *P* < 0.05*, P* < 0.01 and *P* < 0.001 (compared to control group)

^#^, ^##^and ^###^: *P* < 0.05, *P* < 0.01 and *P* < 0.001 (compared to HCC control group)

### Chemoprevention effects of PJ versus PCG on inflammatory status in control and DENA-treated rats

**Fig. 1:**
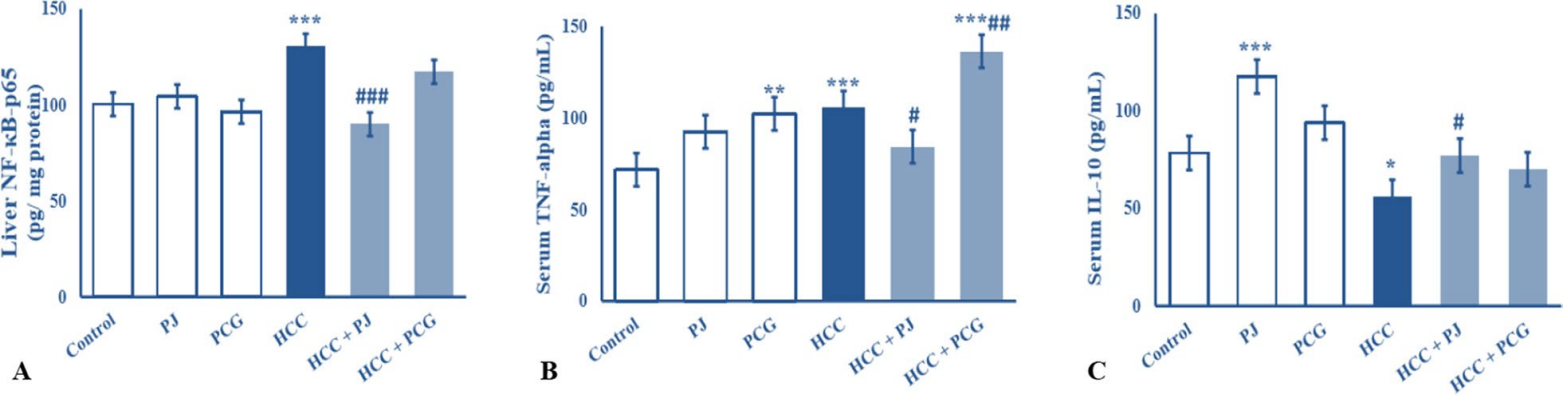
Concentration levels of **A** hepatic NF-κB-p65 (pg/ g protein), **B** serum TNF-α (pg/mL) and **C** serum IL-10 (pg/mL) of non-carcinogenic and carcinogenic male albino rats treated with either pomegranate juice (PJ) or punicalagin (PCG). HCC: hepatocellular carcinogenic-induced group. *, ** and ***: *P* < 0.05, *P* < 0.01 and *P* < 0.001, respectively, compared to control group; #, ## and ###: *P* < 0.05, *P* < 0.01 and *P* < 0.001compared to carcinogenic control group

HCC control group showed a significant increase (*P* < 0.001) in serum TNF-α levels and liver NF-κB-p65 levels as compared with the control group. Oral PJ treatment of DENA-treated rats led to a significant decrease (*P* < 0.05–0.001) in serum TNF-α and liver NF-κB-p65 levels as compared to HCC control rats. However, oral treatment of DENA-treated rats with PCG led to significantly increased serum TNF-α levels (*P* < 0*.*01) and did not significantly affect liver NF-κB-p65 levels (*P* > 0.05) as compared to the HCC control rats. On the contrary, the HCC control group showed a significant decrease (*P* < 0.05) in serum IL-10 levels as compared to the control group. This deterioration in serum IL-10 levels in the HCC control group showed increases with both PJ and PCG treatment. However, the increase was statistically significant (*P* < 0.05) in the PJ group and non-significant (*P* > 0.05) in the PCG group (Fig. 1).

### Chemoprevention effects of PJ versus PCG on expression levels of Caspase-3, Bax, Bcl-2, and Bcl-XL in DENA-treated rats

**Fig. 2:**
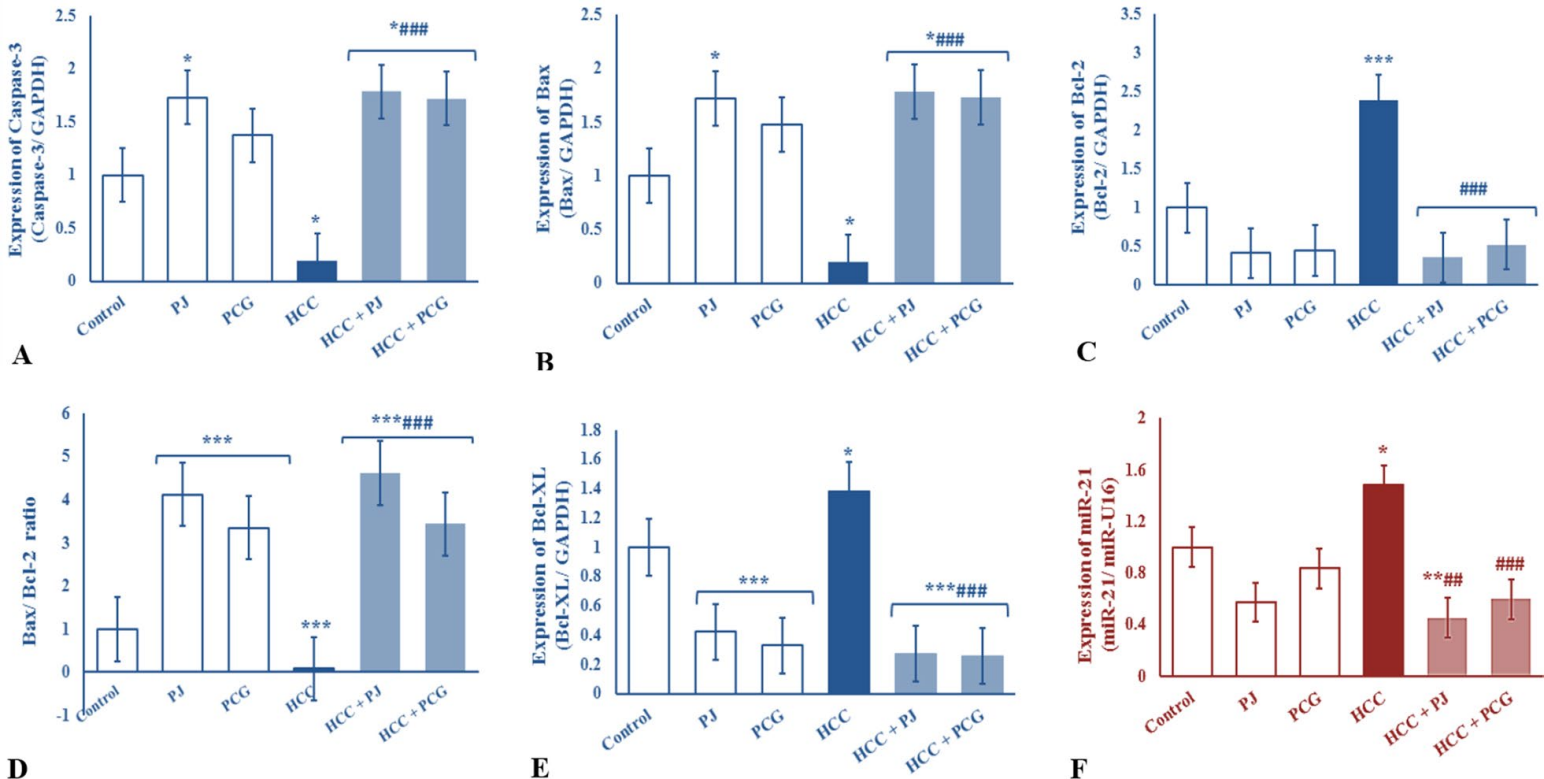
Expression of **A–E** hepatic caspase-3, Bax, Bcl-2, Bax/ Bcl-2 ratio and Bcl-XL mRNA, respectively; **F** Serum circulating miR-21 of non-carcinogenic and carcinogenic male albino rats treated with either pomegranate juice (PJ) or punicalagin (PCG). Values were presented as fold changes after normalization with the expression of a housekeeping miRNA (miR-U16) or a housekeeping gene (GAPDH: glyceraldehyde-3phosphate dehydrogenase) for mRNA. HCC: hepatocellular carcinogenic-induced group. *, ** and ***: *P* < 0.05, *P* < 0.01 and *P* < 0.001, respectively, compared to control group; #, ## and ###: *P* < 0.05, *P* < 0.01 and *P* < 0.001compared to carcinogenic control group

The HCC control group showed a significant downregulation in hepatic caspase-3 and Bax mRNA expression (*P* < 0.05) accompanied by a significant upregulation in the Bcl-XL and Bcl-2 mRNA expression (*P* < 0.05 and *P* < 0.001, respectively). Moreover, the Bax/Bcl-2 ratio showed a significant decrease (*P* < 0.001) in the HCC control group as compared to the control group. However, oral treatment of the DENA-treated rats with either PJ or PCG improved these disturbances in apoptosis as shown by significant upregulation in caspase-3 and Bax mRNA expression (*P* < 0.001), significant downregulation in Bcl-XL and Bcl-2 mRNA expression (*P* < 0.001), and an increased Bax/Bcl-2 ratio as compared to the HCC control rats (Fig. 2A–E).

### Chemoprevention effects of PJ versus PCG on miR-21 expression level in DENA-treated rats

HCC control rats showed a significant upregulation in the expression of serum circulating miR-21 (*P* < 0.05) as compared to control rats. Oral treatment of DENA-treated rats with either PJ or PCG induced a significant downregulation in the expression of serum circulating miR-21 (*P* < 0.001) as compared with the HCC control rats (Fig. 2F).

### Histopathological results

**Fig. 3:**
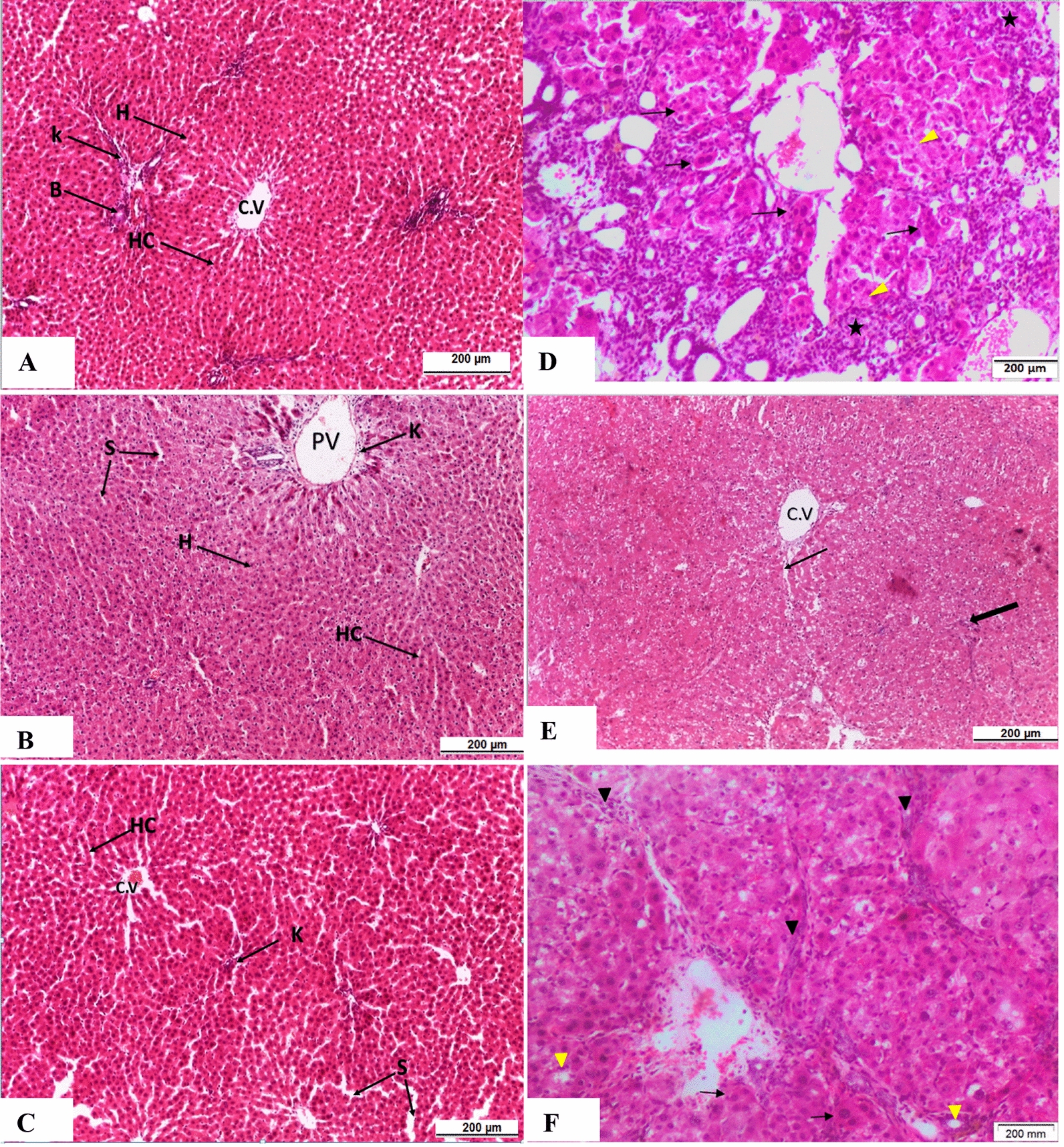
A photomicrograph of liver section of rat from **A** control, **B** PJ, **C** PCG, **D** DENA, **E** DENA and PJ and **F** DENA and PCG (H & E, 200 µm).(CV): Central vein, (H): hepatocyte, (HC): hepatic cords, (K): Kupffer cells, (S): blood sinusoids. Thin arrow: binucleated and multinucleated hepatic cells, Thick arrow: dysplastic cells, Yellow arrow head: vacuolated hepatic cells, Star: inflammatory cell infiltrate, Arrow head: pyknotic hepatocytes

Examination of the liver tissues of rats from the control, PJ-treated, and PCG-treated groups showed a normal pattern of liver architecture with no histopathological alteration. On the other hand, the hepatic tissues from the HCC control group showed distinct alterations, including nodular patterning of the hepatic tissue, dilated central vein, multiple scattered binucleated and multinucleated hepatocytes, vacuolated hepatocytes nuclei with multiple prominent nucleoli, dysplastic cells with increased nuclear cytoplasmic ratios, necrotic hepatocytes, and inflammatory cell infiltration. All of these reported alterations are defined as dysplastic nodules. These high-grade lesions of dysplasia are typically found in cirrhotic livers and have been described as possible progenitor lesions to HCC. In contrast, examination of liver tissue from the DENA-treated group that received PJ showed normal liver architecture with normal central veins and multiple scattered hepatic cells with clear vacuolated cytoplasm and dilated sinusoidal spaces. Liver tissues from the group treated with DENA that received PCG revealed nodular architecture of liver tissue (cirrhotic pattern) with dilated central veins, a few scattered hepatic cells with double nuclei, mild proliferation of interlobular connective tissue, moderate vacuolar degeneration, and mild bile duct hyperplasia (Fig. 3).

## Discussion

HCC is the predominant form of primary liver malignancy. Globally, it is the second-leading cause of cancer mortality, with over 500,000 new cases diagnosed each year [28]. One of the main goals of recent research has sought to determine cancer prevention strategies. To some extent, this can be accomplished by using naturally occurring or synthetic chemoprevention agents that can inhibit or prevent tumor development. Therefore, it is critical to identify agents, assess their efficacy, and elucidate their mechanisms of action. In this respect, it has been revealed that pomegranate and its components have cancer-inhibitory effects on many types of malignancies [29–31]. Accordingly, this study established a model of DENA-induced hepatocarcinogenesis in male albino rats to clarify the role of PJ and PCG in the prevention of HCC development.

Experimental induction of liver cancer in rodents by DENA is one of the most well-characterized animal models of HCC, and it allows for screening of anticancer agents at different stages of neoplastic transformation [32]. DENA-induced rats developed various alterations in liver tissue, defined as dysplastic nodules, which are common in cirrhotic livers. According to an epidemiological survey, more than 80% of HCC cases are associated with advanced liver fibrosis or cirrhosis [33]. Considerable evidence has reported the nodules as precursors of hepatic cancer [34].

DENA-induced preneoplastic foci, as well as preneoplastic and neoplastic nodule formation in rodents, closely resemble the progression of HCC in humans. Cross-species comparison of gene expression revealed that DENA-induced liver tumors in rodents closely resemble a subclass of human HCC, allowing for the extrapolation of possible clinical chemopreventive effects of candidate medicines [32].

Here, the chemopreventive action of PJ in comparison to pure PCG on the development and progression of early preneoplastic lesions in the liver was established in a rat model of hepatocarcinogenesis induced with DENA. The results demonstrate that treatment of DENA-treated rats with PJ/PCG resulted in fewer animals with visible hepatocyte nodules and reduced nodular multiplicity compared to HCC control animals. In a previous literature data it was reported that pomegranate components could prevent malignancies of the skin, breast, lungs, and colon [35]. The present study reported that PJ had a potent chemopreventive effect greater than that of PCG via inhibition of hepatic nodule formation and suppression of nodule growth. These represent important steps for liver cancer chemoprevention.

Liver cancer may produce complex metabolic disturbances resulting in rapid loss of body weight and tissue emaciation [36]. This study reported that the HCC control rats had a significant reduction in body and liver weight, with a significant increase in relative liver weight, as compared to the control group. This was likely attributable to liver carcinogenesis in the HCC control group. These findings converge with many previous studies [37–39]. Whereas treatment of DENA-treated rats with either PJ or PCG significantly increased body weight and decreased relative liver weight when compared to HCC control rats. This finding suggests that PJ and PCG altered the metabolism of energy.

In this study, the HCC control rats showed a significant increase in serum ALAT enzyme activity and a significant decrease in total protein levels, indicating the toxic effect of DENA on liver tissue. These findings align with those of Assar et al*.* and Mokh et al*.* [37, 40], who found that ASAT, ALAT, and ALP activity significantly increased following nitroso compound treatment in rats owing to induced liver damage. The elevation of liver enzymes could be attributed to loss of cell membrane integrity, which allowed them to leak out of the damaged cell into extracellular space, indicating hepatic cellular injury. On the other hand, oral treatment of DENA-treated rats with PJ resulted in a significant decrease in ALAT activity—indicative of hepatic cell function recovery—suggesting that PJ may have a protective effect against DENA-induced liver damage. This hepatoprotective effect of PJ could emerge due to its antioxidant properties that lead to reduced ROS generation and, consequently, reduced membrane permeability and enzyme leakage into the blood [21, 41]. On the other hand, oral treatment of DENA-traeted rats with PCG did not affect serum ALAT activity. According to Lin et al. [42], despite the high antioxidant activity of PCG, it can induce liver damage when taken in high dosages. Although the PCG dose used in this study is less than the doses used in El-Beih et al. [21], we found that it was still not an effective dose.

The decreased serum total protein levels in the HCC control group suggest that synthetic liver function declined and PJ treatment restored serum total protein and synthetic liver function, in line with Husain et al. [41]. This could be due to the antioxidant properties of PJ and could prevent oxidative modification of amino acid chains and ROS-mediated peptide changes caused by DENA.

There is strong evidence that oxidative stress plays a role in the development and progression of cirrhosis, which can lead to liver cancer [4, 5]. ROS and RNS could trigger cellular damage upon exposure to carcinogens, and thus, intoxication of rats with DENA could initiate cell damage through the induction of lipid peroxidation formation, thereby causing cirrhosis and the appearance of preneoplastic lesions that characterize HCC [43]. Further, DENA biotransformation by cytochrome P450 in the rat liver produces ethyl diazonium ions that react with DNA, forming adducts recognized as the initial step in DENA-induced carcinogenesis [44]. Accordingly, HCC control rats showed a significant increase in hepatic levels of MDA and NO, as well as GST activity, which was accompanied by a significant decrease in SOD and CAT enzyme activity, suggesting that DENA injection causes an increase in free radical production, disrupts antioxidant defense systems, and increases ROS, consistent with findings from different cancer models [37, 40]. Interestingly, recent evidence has shown that overactive GSTs play a key role in tumor progression and cancer pathogenesis and are considered a common feature of various human cancers, as they actively participate in tumorigenesis processes such as cell survival, proliferation, and chemoresistance [45, 46]. This was clearly supported by this study, which showed a significant increase in hepatic GST activity in HCC control rats as compared with the control group.

In contrast, this disturbance in the hepatic antioxidant defense system was attenuated in DENA-treated rats that received either PJ or PCG, as hepatic SOD and CAT enzyme activity showed significant increases in rats administered PJ. However, CAT activity improvement in the DENA-treated rats that received PCG was statistically non-significant. This improvement in hepatic antioxidants was coupled with a significant decrease in the hepatic levels of MDA and NO as compared to the HCC control group. Notably, the present data showed that treatment with either PJ or PCG led to a significant decrease in hepatic GST activity compared to the HCC control group. Thus, PJ or PCG could contribute to the development of GST inhibitors for cancer treatment. Pomegranate could reverse the progression of pathological lesions via its strong antioxidant capacity, as it has potent free-radical scavenging properties [47]. PJ may reduce the toxicity of heterocyclic aromatic amine and prevent the hepatocarcinogenic effect of nitrosamines. These findings support various studies documenting the hepatoprotective effects of pomegranate against chemically induced hepatocarcinogenesis and its ability to inhibit lipid peroxidation and oxidative damage [32]. In addition, Aloqbi et al*.* [48] reported that PCG contains 16 phenolic hydroxyls per molecule, whereas PJ contains higher concentrations of anthocyanins than tannin components, perhaps explaining why PJ has greater antioxidant capacity, as compared to PCG, in reducing stable radicals to a non-radical state.

Active NF-κB is frequently implicated in various malignancies and in inflammation. Excessive generation of ROS may activate the NF-κB pathway, the most prominent pathway involved in the inflammation-fibrosis-cancer axis, and trigger release of IĸBs, leading to NF-κB nuclear translocation. Thus, it may enhance the release of pro-inflammatory mediators, thereby leading to inflammation. Inflammation could trigger disruption of the hepatic microenvironment, creating an adverse oncogenic field. Thus, inflammatory signaling pathways may be considered a target for cancer prevention [47, 49, 50]. A few recent studies have examined the molecular mechanisms that link inflammation to carcinogenesis. Considering that NF-κB-p65 has been reported to be related to HCC progression, Xu et al*.* [1] revealed that NF-κB-p65 was activated and that the translocation of its phosphorylated isoform into the nucleus was markedly increased after DENA and TNF-α treatment. Similarly, the data of this study showed that oxidative stress induced by DENA and initiated hepatocarcinogenesis in rats affected the inflammatory response of liver cells by triggering NF-κB-p65 protein and elevating serum TNF-α, in conjunction with reduced IL-10 levels, as compared to the levels observed in the control rats. This aligns with findings suggesting that NF-κB is the most important transcriptional factor, which directly controls pro-inflammatory cytokine cellular production [16]. TNF-*α*, which plays a major role in inflammation and has also been shown to be an accelerant of cell proliferation, is a powerful NF-κB-activating factor whose expression is regulated by NF-κB [51]. Driessler et al. [52] suggested that anti-inflammatory properties of IL-10 may act by suppressing the expression of pro-inflammatory cytokines via inhibiting NF-κB. However, it seems that the balance between TNF-ɑ and IL-10 is critical to the development of HCC [53]. Thus, elevated TNF-α associated with low levels of IL-10 were shown to be associated with increased risk of HCC development.

In this study, DENA-treated rats that received PJ showed an improvement in the inflammatory state as demonstrated by the inhibitory effects of PJ on NF-κB-p65 production, which was associated with reduced serum TNF-α and increased IL-10 concentrations. While, DENA-treated rats that received PCG showed no effect on NF-κB-p65 and IL-10 levels. As NF-κB is an oxidant-sensitive transcription factor, PJ could inhibit the activation of NF-κB by suppressing oxidative stress. This is in alignment with the previous report showing that pomegranate extract inhibits nuclear translocation and activation of the NF-κB-p65 protein in human chondrocytes [54]. Further, in their review paper, Syed et al. [31] have presented literature data on therapeutic action of PJ via its ability to downregulate NF-κB expression by suppressing the IκBα kinase (IKK) activation, which stops cell growth and induces apoptosis. Moreover, PJ treatment reduced TNF-α level, which resulted in NF-κB inhibition and subsequent inhibition of cell proliferation. This reduction in TNF-α was associated with a significant elevation in IL-10 levels, indicating the potent anti-inflammatory effect of PJ in the initiation of HCC in DENA-treated rats.

Oxidative stress and inflammation are thought to work as the primary initiators of apoptosis, which is controlled by either mitochondrial (intrinsic) or death receptor-mediated (extrinsic) signaling pathways. Anti-apoptotic proteins (Bcl-2 and Bcl-XL) and pro-apoptotic proteins (Bax and caspase-3) are essential components of mitochondrial-mediated apoptosis [55]. When Bcl-2 protein mRNA levels drop and Bax protein levels rise, mitochondrial membrane breakdown occurs, and this allows cytochrome *C* to leak into the cytosol. Then, a cascade of reactions occurs, including cytochrome binding to Apaf-1, resulting in activation of caspase-9 and caspase-3 [47]. Various studies suggest that DENA could trigger both apoptotic pathways initiated by oxidative stress in the liver [56, 57]. In contrast, our findings indicate upregulation in Bcl-2 and Bcl-XL and downregulation in Bax and caspase-3 mRNA levels in the livers of rats intoxicated with DENA. In rats, DENA mediated the anti-apoptotic effect via downregulation of caspase-3 mRNA levels and a decreased Bax/Bcl-2 ratio. Exposure to DENA is associated with hepatocellular accumulation of ROS [58], which in turn could trigger apoptosis in the liver [59, 60]. Chavda et al. [61] published literature evidence in their review work that the oxidative stress can cause cell apoptosis only at high concentrations. Thus, ROS levels play a critical role in either promoting tumorigenesis or causing apoptosis.

The present results show that administering either PJ or PCG to DENA-treated rats reversed the progression of the pathological lesions through activation of the apoptosis process, as it significantly downregulated Bcl-2 and Bcl-XL mRNA and upregulated the expression of Bax and caspase-3 mRNA, in line with Ghani et al. [62], who found that pomegranate could trigger apoptosis via the intrinsic pathway through cytochrome *C* release and caspase-3 activation. PCG could render the hepatocytes more susceptible to TNF-α-induced (extrinsic) apoptosis, which is indirectly related to the depletion of hepatocytes GSH conjugates, resulting from decreased GST activity in the livers of DENA-treated rats that received PCG [46].

MiRNAs have been implicated in cancer development, and their expression in biological fluids offers considerable potential as nucleic acid markers in cancer diagnosis and prognosis. This study revealed that circulating oncomir miR-21 expression was significantly increased in HCC control rats as compared to the control group. This finding is in accordance with the previous work revealing miR-21 overexpression in DENA-infused rats and suggested that serum miR-21 could be employed as an early diagnostic molecular marker in hepatocarcinogenesis [63]. It was found that miR-21 usually has multiple targets and thus can modulate multiple molecular mechanisms of biological processes in HCC models [64]. Zhang et al. [65] found that the role of miR-21 in carcinogenesis might be linked to its effect on ROS levels via targeting SOD3 and TNFα. Moreover, miR-21 overexpression affects cell proliferation and inhibits apoptosis by targeting Bax downregulation and Bcl-2 upregulation [66].

**Fig. 4:**
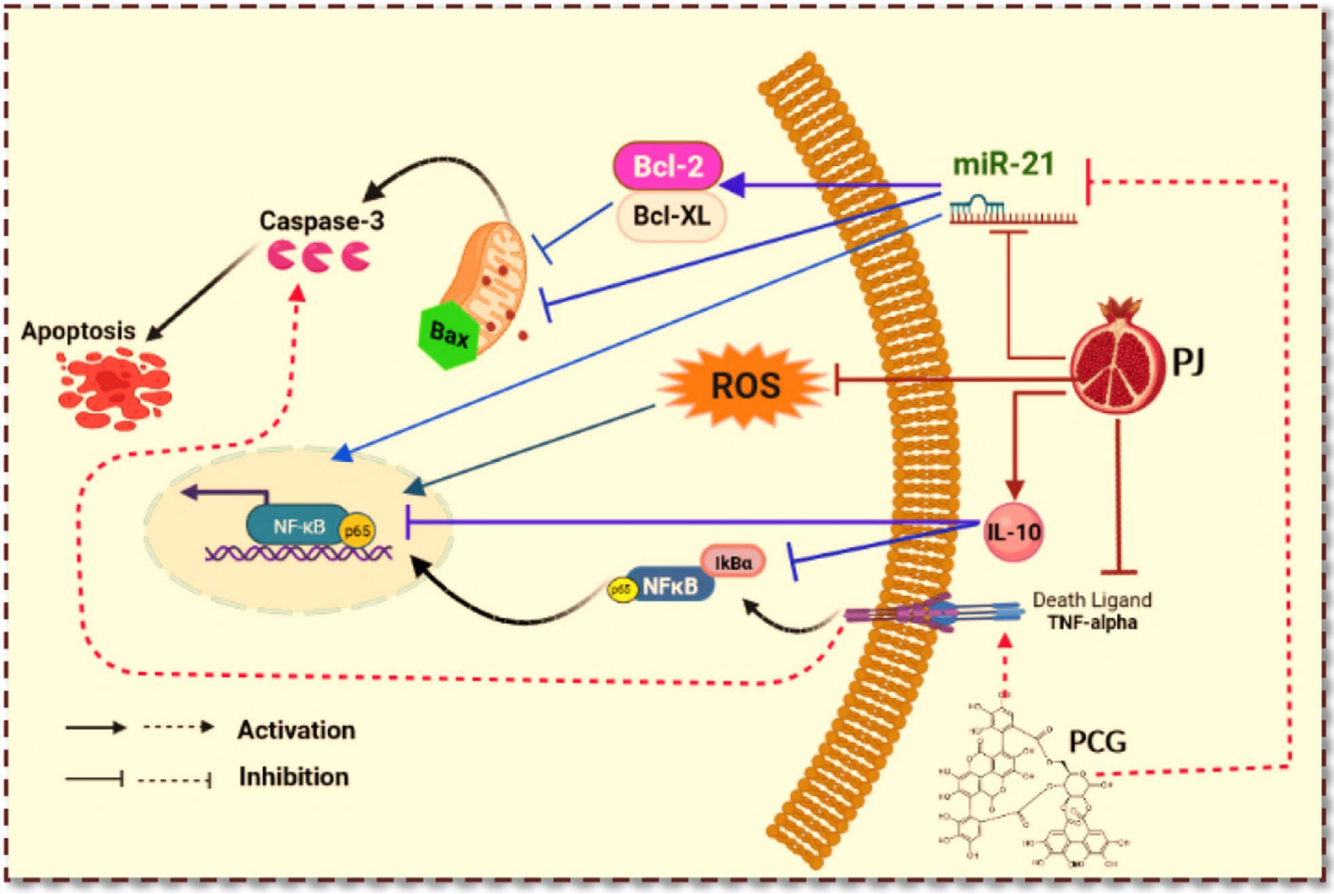
Schematic diagram summarizes the pomegranate juice (PJ) and punicalagin (PCG) mediated molecular mechanisms contributing to chemoprevention of hepatocellular carcinogenesis. Abbreviations: Bax: Bcl-2-associated X protein; Bcl-2: B-cell lymphoma 2; Bcl-XL: B-cell lymphoma-extra-large; IL-10: interleukin-10; NF-κB: Nuclear Factor Kappa-B; ROS: reactive oxygen species; TNF-α: tumour necrosis factor- alpha

In contrast, the current data demonstrate that oral administration of either PJ or PCG significantly downregulated miR-21 expression in comparison to the DENA-treated group, in line with studies reporting a downregulation effect of pomegranate on miR-21 expression [67, 68]. Given the current data, the downregulation effect of PJ or PCG on miR-21 expression may be attributable to their anti-inflammatory effect via the NF-κB-p65/TNF-α pathway, which can affect miR-21 expression (Fig. 4). This aligns with previous studies demonstrating that miR-21 is a type of NF-κB-dependent miRNA that shows increases in response to cytokines and inflammation, specifically via activation of NF-κB signaling, indicating the interplay between miR-21 and NF-κB in cancer [69, 70].

## Conclusion

The present study revealed the chemopreventive role of PJ and PCG against hepatocarcinogenesis and elucidated their possible mechanisms of action. Both of PJ and PCG downregulated miR-21 expression and triggered apoptosis related genes expression. However; PJ was superior to pure PCG in improving the hepatic antioxidant defense state and the inflammatory status, which increases its chemoprevention effectiveness against DENA-induced hepatocellular carcinogenesis. So, further studies are required to provide the appropriate dose–response of PCG on HCC.

## Data Availability

Not applicable.

## Abbreviations

ALAT: Alanine aminotransferase
ALP: Alkaline phosphatase
ANOVA: Analysis of variance
Apaf-1: Apoptotic protease activating factor 1
ASAT: Aspartate aminotransferase
Bax: Bcl-2 Associated X-protein
Bcl-2: B-cell lymphoma 2
Bcl-XL: B-cell lymphoma-extra large
CAT: Catalase
cDNA: Complementary deoxyribonucleic acid
Ct: Cycle threshold
DENA: Diethylnitrosamine
ELISA: Enzyme-linked immunosorbent assay
GAPDH: Glyceraldehyde-3-phosphate dehydrogenase
GSH: Glutathione reduced
GST: Glutathione S-transferase
HCC: Hepatocellular carcinoma
HPLC: High-performance liquid chromatography
IĸBs: Inhibitor κBs
IKK: IκBα kinase
IL-10: Interleukin-10
I.P.: Intraperitoneal
MDA: Malondialdehyde
mRNA: Messenger ribonucleic acid
NF-κB-p65: Nuclear factor-κB-p65
NO: Nitric oxide
PCG: Punicalagin
PJ: Pomegranate juice
qPCR: Quantitative real time polymerase chain reaction
RNA: Ribonucleic acid
RNS: Reactive nitrogen species
ROS: Reactive oxygen species
RT: Room temperature
SEM: Standard error of the mean
SOD: Superoxide dismutase
TNF-α: Tumour necrosis factor-α
WHO: World health organization
